# A psychological stressor conveyed by appetite-linked neurons

**DOI:** 10.1126/sciadv.aay5366

**Published:** 2020-03-18

**Authors:** Eun Jeong Lee, Naresh K. Hanchate, Kunio Kondoh, Ai Phuong S. Tong, Donghui Kuang, Andrew Spray, Xiaolan Ye, Linda B. Buck

**Affiliations:** Fred Hutchinson Cancer Research Center, 1100 Fairview Avenue North, A3-020, Seattle, WA 98109, USA.

## Abstract

Mammals exhibit instinctive reactions to danger critical to survival, including surges in blood stress hormones. Hypothalamic corticotropin-releasing hormone neurons (CRHNs) control stress hormones but how diverse stressors converge on CRHNs is poorly understood. We used sRNA profiling to define CRHN receptors for neurotransmitters and neuromodulators and then viral tracing to localize subsets of upstream neurons expressing cognate receptor ligands. Unexpectedly, one subset comprised POMC (proopiomelanocortin)–expressing neurons in the arcuate nucleus, which are linked to appetite suppression. The POMC neurons were activated by one psychological stressor, physical restraint, but not another, a predator odor. Chemogenetic activation of POMC neurons induced a stress hormone response, mimicking a stressor. Moreover, their silencing markedly reduced the stress hormone response to physical restraint, but not predator odor. These findings indicate that POMC neurons involved in appetite suppression also play a major role in the stress hormone response to a specific type of psychological stressor.

## Teaser

New studies show that POMC neurons linked to appetite suppression also play a key role in stress hormone responses.

## INTRODUCTION

Corticotropin-releasing hormone neurons (CRHNs) in the paraventricular nucleus (PVN) of the hypothalamus are master controllers of blood stress hormones (*1*, *2*). In mice, they induce increases in stress hormones in response to predator odors, injury, and a variety of other external and internal stressors, including psychological stress associated with physical restraint (*3*–*6*). The stress hormone increases recruit multiple tissue systems to coordinate appropriate responses to external and internal threats.

Classical neurophysiological and neuroanatomical studies have provided numerous insights into CRHNs and stress hormone responses to diverse stimuli (*1*–*5*). More recent viral tracing studies revealed that mouse CRHNs receive direct synaptic input from neurons in 31 different brain areas (*7*). However, detailed information on how this complexity of neural connections mediates responses to diverse stressors remains to be explored.

To investigate this question, we sought to identify molecular markers that could define subsets of neurons upstream of CRHNs in different areas. The information obtained would lay a foundation for the molecular interrogation of the upstream circuits for the stress responses in which they are involved and provide tools with which to dissect their functions. The strategy we devised was to identify receptors on CRHNs for neurotransmitters and neuromodulators and then determine the locations of upstream neurons expressing ligands of those receptors. We call this method RAMUN (receptor-assisted mapping of upstream neurons) (fig. S1).

Using RAMUN, we identified a large number and variety of receptors expressed by CRHNs. With viral tracing, we then identified neuropeptide ligands of a number of receptors in upstream neurons in specific brain areas. One subset consisted of proopiomelanocortin (POMC) neurons in the hypothalamic arcuate nucleus, which have been implicated in appetite suppression and satiety (*8*, *9*). Functional studies revealed that POMC neurons can be activated by physical restraint, but not another psychological stressor, a predator odor. Chemogenetic experiments further showed that, like restraint, activation of POMC neurons can induce a stress hormone increase. Furthermore, silencing of POMC neurons caused a marked decrease in the stress hormone response to restraint but had no effect on the stress hormone response to predator odor. These findings indicate that arcuate nucleus POMC neurons linked to satiety also play an important and selective role in the stress hormone response to one type of psychological stressor, physical restraint, but not another, a predator odor. These studies also lay the groundwork for future studies of the roles played by molecularly identifiable subsets of neurons upstream of CRHNs in stress responses to other internal and external stressors.

## RESULTS

### CRHNs express a variety of receptors for neurotransmitters and neuromodulators

We first analyzed the transcriptomes of single isolated CRHNs to identify receptors that they express for neurotransmitters and neuromodulators. We crossed CRH-IRES-Cre (CRH-Cre) mice (*10*) with Ai14 reporter mice (*11*), which have Cre-dependent expression of tdTomato. We then manually isolated single fluorescent cells from dissociated PVN tissue and conducted single-cell RNA sequencing (RNA-seq) to define the transcriptomes of individual isolated neurons (*12*).

**Fig. 1:**
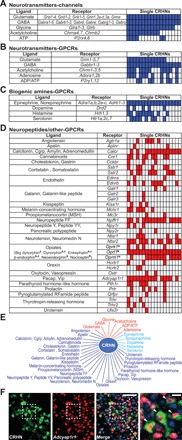
CRHNs have a variety of receptors for neurotransmitters and neuromodulators. (**A** to **D**) Neurotransmitter and neuromodulator receptor genes detected in transcriptome data from at least two manually isolated CRHNs. Boxes on the right indicate individual CRHNs. Blue boxes indicate CRHNs that express one or more receptors of a given type (A to C), and red boxes indicate those expressing individual receptors. Receptors for neurotransmitters include ligand-gated ion channels (A) and GPCRs (B). CRHNs also have GPCRs for biogenic amines (C) and a variety of neuropeptides and cannabinoids (D). See fig. S2 for details of receptor gene expression shown in (A) to (C). Each type of opiate is marked with superscripts (a) to (c) corresponding to its receptor on the right (Cgrp, calcitonin gene–related peptide; MSH, melanocyte-stimulating hormone; Pacap, pituitary adenylate cyclase–activating peptide; Vip, vasoactive intestinal polypeptide). (**E**) A diagram of ligands whose receptors are expressed in CRHNs. Red, neurotransmitter; light blue, biogenic amine; dark blue, neuropeptide or cannabinoid. (**F**) Representative photographs of a PVN section from CRH-Cre mice crossed with Ai6-ZsGreen reporter mice, costained with anti-GFP antibodies to detect CRHNs (green) and an *Adcyap1r1* riboprobe (red), and counterstained with DAPI (blue). A higher magnification of the boxed area showing CRHNs colabeled for *Adcyap1r1* is shown on the right. Arrowheads indicate colabeled cells. Scale bars, 100 μm (left) and 20 μm (right).

These experiments revealed that CRHNs express a large variety of neurotransmitter and neuromodulator receptors (Fig. 1 and fig. S2). Consistent with previous studies (*13*, *14*), these include ligand-gated ion channels for glutamate and γ-aminobutyric acid (GABA), the major excitatory and inhibitory neurotransmitters in the brain, respectively. We also identified ion channels gated by other “fast neurotransmitters,” such as acetylcholine, glycine, and adenosine triphosphate (ATP) (Fig. 1A and fig. S2A). In addition, we found G protein–coupled receptors (GPCRs) for glutamate, GABA, acetylcholine, adenosine, and adenosine diphosphate (ADP)/ATP, which allow these neurotransmitters to more indirectly modulate neuronal activity (Fig. 1B and fig. S2B).

These experiments also identified a large number of other GPCRs for neuromodulators, which may either enhance or dampen CRHN excitability. These include receptors for the neuromodulatory biogenic amines, epinephrine, norepinephrine, dopamine, histamine, and serotonin as well as receptors for a variety of different neuropeptides (Fig. 1, C and D, and fig. S2C). Together, we found 41 ligand-gated ion channel subunits and 64 GPCRs, which include receptors for seven neurotransmitters and five biogenic amines as well as 33 receptors for neuropeptides and cannabinoids (Fig. 1E). We also analyzed previous sequencing data for single hypothalamic neurons available in the Gene Expression Omnibus (GSE74672) (*15*, *16*). In the 86 CRH-expressing neurons that we found in those data, we identified 80 GPCRs, 54 of which were also identified in our experiments. Together, these findings suggest that CRHN excitability and stress hormone responses can be modulated by a diverse array of signaling molecules expressed by upstream neurons.

We verified expression of selected receptors in CRHNs by crossing CRH-Cre and Ai6-ZsGreen reporter mice (*11*) and then costaining brain sections with receptor-specific riboprobes and anti–green fluorescent protein (GFP) (ZsGreen) antibodies to detect CRHNs (Fig. 1F and fig. S3A). The percentage of CRHNs expressing individual receptors varied, with different receptor probes colabeling from 6.2 to 58.8% of CRHNs (fig. S3B). Variation was also seen in the transcriptome data (Fig. 1, A to D), suggesting that there may be subsets of CRHNs that express different constellations of receptors and are thus subject to regulation by different upstream signaling molecules.

### Neuropeptide ligands of CRHN receptors localize to specific subsets of upstream neurons

**Fig. 2:**
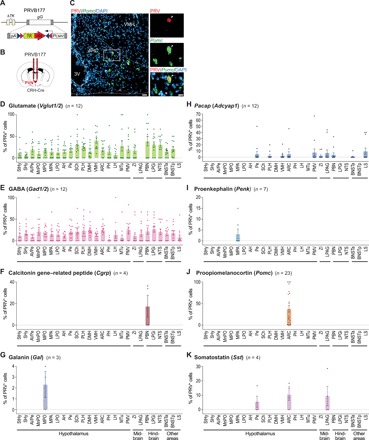
Ligands of different CRHN receptors identify subsets of upstream neurons in specific brain areas. (**A** and **B**) PRVB177 (A) was injected into the PVN of CRH-Cre mice to infect CRHNs (B). (**C** to **K**) On d3pi, brain sections were costained with anti-HA antibodies to label PRV^+^ neurons and riboprobes to detect expression of specific neurotransmitters or neuromodulators recognized by CRHN receptors. (C) Photographs show a section through the ARC containing a PRV^+^ neuron (red) colabeled for *Pomc* mRNA (green). A higher magnification of the boxed area with the cell colabeled for PRV and *Pomc* (arrowheads) is shown on the right. VMH, ventromedial hypothalamic nucleus; 3V, third ventricle. Scale bars, 50 μm (left) and 10 μm (right). (D to K) Graphs show percentage of PRV^+^ neurons colabeled with riboprobes for different neurotransmitters and neuropeptides in different brain areas, which are indicated below. Numbers of animals are indicated in parentheses. Column heights indicate means, and error bars indicate SEM. Dots in the same column indicate different animals. See Materials and Methods for full names of abbreviated brain areas. The location of each brain area is indicated on schematic brain sections in fig. S4A. Glutamate and GABA were expressed in upstream neurons in most areas, whereas neuropeptides were expressed in upstream neurons in only specific areas.

We next investigated the locations of neurons upstream of CRHNs that express ligands for receptors on CRHNs. Using CRH-Cre mice, we infected CRHNs with PRVB177, a pseudorabies virus with Cre-dependent expression of hemagglutinin (HA) fused to thymidine kinase (TK) (Fig. 2, A and B) (*7*). This virus travels retrogradely across synapses in a time-dependent manner and labels directly presynaptic neurons on day 3 post-infection (d3pi) (fig. S4A) (*7*). It cannot be absolutely excluded that some PRVB177-infected neurons on d3pi are two synapses upstream, although this appears unlikely. On d3pi, we costained brain sections with anti-HA antibodies to visualize PRV-infected neurons and riboprobes specific for individual ligands or markers (e.g., transporters or biosynthetic enzymes) indicative of their expression (Fig. 2C and fig. S4B).

Unexpectedly, most brain areas containing upstream neurons (24 of 26 areas) showed some PRV^+^ neurons colabeled for markers of excitatory glutamatergic neurons (*Vglut1/2*) and some colabeled for inhibitory GABAergic neuron markers (*Gad1/2*) (Fig. 2, D and E). Thus, most of the upstream areas may be able to transmit either excitatory or inhibitory signals to CRHNs. While the excitatory inputs could activate CRHNs in response to stressors, the role of inhibitory inputs is less obvious. They could conceivably provide tonic inhibitory input to CRHNs under basal conditions (*3*, *5*, *13*, *14*) or be involved in the blocking of stress hormone responses, as seen with certain odors (*17*, *18*).

In sharp contrast to glutamate and GABA, neuropeptide ligands of CRHN receptors were selectively expressed in upstream neurons in specific brain areas (Fig. 2, F to K, and fig. S4C). For example, galanin (*Gal*), proenkephalin (*Penk*), and POMC (*Pomc*) were each detected in upstream neurons in only one area of the hypothalamus, which differed for the three neuropeptides [*Gal* in MPO (medial preoptic nucleus), *Penk* in MPA (medial preoptic area), and *Pomc* in ARC (arcuate hypothalamic nucleus)]. Another neuropeptide, calcitonin gene–related peptide (*Cgrp*), was seen in upstream neurons in only one hindbrain area. Yet another neuropeptide, *Pacap* or *Adcyap1*, was detected in PRV^+^ neurons in a number of hypothalamic areas and other areas, but not all areas.

It cannot be excluded that additional areas contain upstream neurons expressing the tested ligands of CRHN receptors, because animals varied in the number of PRV^+^ neurons in different areas (fig. S5, A and B) and the proportion of those expressing specific receptor ligands (Fig. 2, D to K). Despite this variability, the data indicate that upstream neurons in different brain areas can use different neuropeptides to communicate with CRHNs and potentially enhance or dampen their excitability.

These findings represent a substantial step in the elucidation of a molecular map of CRHN neural circuits. They provide molecular tools that can be used to dissect the functions of neurons in different brain areas in response to different stressors.

### Individual stressor signals may reach CRHNs via parallel pathways

What are the locations of upstream neurons that convey signals about specific types of stressors? Is information about different stressors provided to CRHNs via upstream neurons in the same area or neurons in different areas, and could activating signals induced by a single stressor reach CRHNs through a single channel—or multiple parallel channels?

**Fig. 3:**
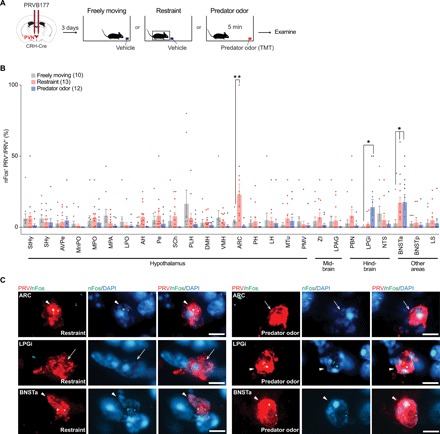
Stressors activate upstream neurons in specific brain areas. (**A**) CRHNs were infected with PRVB177, and animals were exposed on d3pi to filter paper containing water while freely moving or physically restrained or to filter paper containing the predator odor TMT. (**B**) The graph shows the percentage of PRV^+^ cells in different brain areas that were colabeled for the neuronal activity marker nFos following the exposures shown in (A). Numbers of animals are shown in parentheses. Column heights indicate means, and error bars indicate SEM. Dots in each column indicate different animals. *P < 0.05 and **P < 0.01, Kruskal-Wallis test with post hoc Dunn’s test. Significant activation of upstream neurons was seen in only three areas. (**C**) Images show sections of ARC, BNSTa, or LPGi containing a PRV^+^ neuron (red) colabeled for nFos (green) or not after exposure to physical restraint or predator odor. Arrowheads indicate colabeled cells, and arrows indicate PRV^+^ nFos^−^ cells. Blue, DAPI. Scale bars, 10 μm.

To explore these questions, we examined the locations of upstream neurons activated by two different psychological stressors: the fox predator odor TMT (2,5-dihydro-2,4,5-trimethylthiazoline) (*19*, *20*) and physical restraint (*6*, *21*). We infected CRHNs with PRVB177 and, on d3pi, exposed animals to filter paper containing TMT or to physical restraint (Fig. 3A). As a control, freely moving animals were exposed to filter paper containing water. Brain sections were then costained with HA antibodies (PRV) and a riboprobe for the neuronal activity marker, nuclear *c-Fos* mRNA (nFos) (*22*).

Three areas showed significant stressor-induced activation of upstream neurons: BNSTa (anterior bed nucleus of the stria terminalis), ARC, and LPGi (lateral paragigantocellular nucleus). The ARC showed significant activation of PRV^+^ neurons by restraint, but not TMT, and the LPGi by TMT, but not restraint (Fig. 3, B and C, and fig. S5C). In contrast, the BNSTa showed significant activation of PRV^+^ neurons by both restraint and predator odor (Fig. 3, B and C, and fig. S5C). These results indicate that neurons upstream of CRHNs in ARC can be activated by physical restraint, those in LPGi can be activated by predator odor, and those in the BNSTa can be activated by either restraint or predator odor.

Because of the low and variable numbers of PRV^+^ neurons in different areas (fig. S5, A and B), these experiments cannot exclude the possibility that upstream neurons in additional brain areas are stimulated by restraint or predator odor. Nevertheless, these results indicate that more than one area can convey signals to CRHNs about a specific stressor and that a single brain area can transmit signals to CRHNs about more than one stressor. They also suggest that some brain areas may transmit signals to CRHNs about one stressor, but not another.

### ARC *Pomc*^+^ neurons are required for the stress hormone response to one psychological stressor, but not another

The studies to locate CRHN receptor ligands in upstream neurons indicated that about 40% of PRV^+^ neurons upstream of CRHNs in ARC expressed *Pomc* (Fig. 2J and fig. S4C). Restraint was previously found to induce *c-Fos* in ARC POMC neurons in rat (*23*). However, the presence of *Pomc*^+^ neurons upstream of CRHNs was not known at the time, and a connection between *Pomc*^+^ ARC neurons and CRHNs was not explored.

**Fig. 4:**
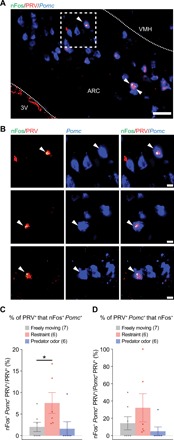
Physical restraint activates POMC neurons upstream of CRHNs in ARC. (**A**) The photograph shows ARC PRV^+^ neurons colabeled for *Pomc* and nFos (arrowheads). Locations of adjacent third ventricle and VMH are indicated. The boxed area is shown in the top row in (B). Scale bar, 100 μm. (**B**) Photographs show single ARC PRV^+^ neurons colabeled for *Pomc* and nFos (arrowheads). Each row shows a section from a different animal. The top row shows the boxed area in (A). Scale bars, 10 μm. (**C**) The graph shows the percentage of ARC PRV^+^ cells colabeled for nFos and *Pomc* in freely moving animals and those exposed to restraint or predator odor. Numbers of animals are shown in parentheses. Dots indicate different animals. Column heights indicate means, and error bars indicate SEM. Mann-Whitney test, *P < 0.05. Restraint induced a significant increase in colabeled neurons, but predator odor did not. (**D**) The graph shows the percentage of ARC PRV^+^*Pomc*^+^ cells colabeled for nFos in the experiments shown in (C). Animals exposed to restraint showed a higher proportion of colabeled cells than those exposed to predator odor or freely moving control animals.

To examine the responses of ARC *Pomc*^+^ neurons to stressors, CRHNs were infected with PRVB177, and on d3pi, mice were exposed to stressors or water, as above (Fig. 3A). Sections through the ARC were then costained with anti-HA antibodies (PRV) and riboprobes for nFos and *Pomc* (Fig. 4, A and B).

In the freely moving, restraint, and predator odor categories, respectively, three of seven, six of six, and three of six animals showed nFos^+^ PRV^+^ neurons in ARC, and three of seven, six of six, and one of six had nFos^+^PRV^+^*Pomc^+^* neurons in ARC (Fig. 4, C and D, and fig. S5, D and E). Restraint induced a significant increase in ARC PRV^+^ cells labeled for nFos and *Pomc* (a 3.2-fold increase compared to freely moving controls), whereas predator odor did not (Fig. 4C and fig. S5D). Restraint, but not predator odor, also induced an increase in *Pomc*^+^PRV^+^ cells labeled for nFos (a 1.7-fold increase compared to freely moving controls) (Fig. 4D). Moreover, a high proportion of restraint-activated upstream neurons in ARC expressed *Pomc* (68.7 ± 14.7% of nFos^+^PRV^+^ ARC neurons were costained for *Pomc*) (fig. S5E). These results suggest that ARC POMC neurons upstream of CRHNs can be stimulated by physical restraint, but not by the predator odor TMT.

**Fig. 5:**
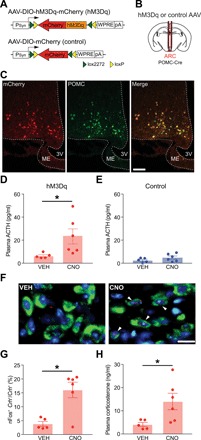
Chemogenetic activation of POMC neurons induces stress hormone increase. (**A** and **B**) The effect of POMC neuron activation was examined by using an AAV encoding hM3Dq-mCherry (or mCherry alone as a control) (A) to infect POMC neurons in POMC-Cre mice (B). (**C**) Images show a section through ARC immunostained for mCherry (red) and POMC after injection of AAV-DIO-hM3Dq-mCherry (A) into the ARC. ME, median eminence. This section has a high proportion of POMC neurons infected with AAV. All mCherry^+^ cells are POMC^+^, but some POMC^+^ neurons lack mCherry. Scale bar, 100 μm. (**D** and **E**) Graphs show concentrations of plasma ACTH following CNO or vehicle (VEH) injection of animals with POMC neurons infected with the AAV encoding hM3Dq-mCherry (D) or mCherry alone (Control) (E). Individual dots indicate different animals. Column heights indicate means, and error bars indicate SEM. Unpaired *t* test, *P < 0.05. CNO induced a significant increase in ACTH in animals infected with the hM3Dq AAV, but not the control virus, indicating that POMC neuron activation increases plasma ACTH. (**F**) PVN neurons colabeled for *Crh* (green) and nFos (red) after injection of vehicle or CNO into mice expressing hM3Dq in ARC POMC neurons. Blue, DAPI. Scale bar, 10 μm. POMC neuron activation induced nFos in many CRHNs (*Crh*^+^). (**G**) Percentage of PVN *Crh*^+^ neurons colabeled for nFos in mice treated as in (D). Individual dots indicate different animals. Column heights indicate means, and error bars indicate SEM. Mann-Whitney *U* test, *P < 0.05. CNO induced a significant increase in activated (nFos^+^) CRHNs in animals infected with the hM3Dq AAV, indicating that POMC neuron activation stimulates CRHNs. (**H**) Plasma corticosterone concentration after mice expressing hM3Dq in ARC POMC neurons were injected with vehicle or CNO. Individual dots indicate different animals. Column heights indicate means, and error bars indicate SEM. Unpaired *t* test, *P < 0.05. CNO induced a significant increase in corticosterone in animals infected with the hM3Dq AAV, indicating that POMC neuron activation increases plasma corticosterone.

These findings raised the possibility that ARC *Pomc*^+^ neurons might play a part in the stress hormone response to physical restraint, but not predator odor. To explore this idea, we first asked whether chemogenetic activation of ARC *Pomc*^+^ neurons can induce a stress hormone increase. We injected the adeno-associated virus (AAV) AAV-DIO-hM3Dq-mCherry (*24*) into the ARC of mice with Cre expressed in POMC neurons (POMC-Cre mice) (Fig. 5, A to C) (*25*). This virus has Cre-dependent expression of mCherry fused to hM3Dq, a receptor that depolarizes neurons upon binding to clozapine-N-oxide (CNO) (*26*). Control mice received the control virus AAV-DIO-mCherry (*27*). We then injected animals with CNO or vehicle and measured plasma levels of the stress hormone, adrenocorticotropic hormone (ACTH).

Chemogenetic activation of POMC neurons induced a marked increase in plasma ACTH (Fig. 5D). Animals with POMC neurons infected with AAV-hM3Dq showed a 4.8-fold increase in ACTH after treatment with CNO compared to vehicle. In contrast, animals infected with the control virus showed similar levels of ACTH in response to CNO and the vehicle control (Fig. 5E). Consistent with these results, activation of POMC neurons also induced nFos in PVN CRHNs (Fig. 5, F and G) [but not PVN arginine vasopressin (AVP) neurons (fig. S6)], and it caused plasma increases in the stress hormone corticosterone (Fig. 5H). Additional analyses showed that mCherry^+^ (AAV infected) neurons were present only in ARC (fig. S7A). These results indicate that activation of ARC POMC neurons, like exposure to a stressor, can induce a stress hormone increase by stimulating CRHNs in the PVN.

**Fig. 6:**
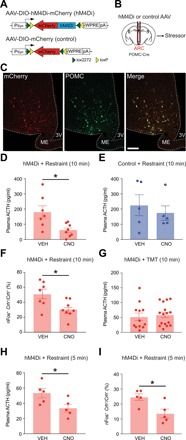
Chemogenetic silencing of POMC neurons inhibits the stress hormone response to restraint. (**A** and **B**) The effect of POMC neuron silencing was investigated by infecting POMC neurons of POMC-Cre mice with an AAV encoding hM4Di-mCherry or a control AAV encoding mCherry alone. (**C**) Immunostaining of an ARC section for mCherry (red) and POMC (green) after injection of AAV-DIO-hM4Di-mCherry into the ARC. A high proportion of POMC neurons are infected with AAV in this section. All mCherry^+^ cells are POMC^+^, but some POMC^+^ neurons lack mCherry. Scale bar, 100 μm. (**D** and **E**) Graphs show plasma ACTH concentrations in animals that received the silencing (hM4Di) or control AAV and then were exposed to restraint for 10 min after injection with CNO or vehicle. Dots indicate individual animals. Column heights indicate means, and error bars indicate SEM. POMC neuron silencing caused a significant reduction in the ACTH response to restraint (D). Infection of POMC neurons with the control virus did not affect the response to restraint (E). Unpaired *t* test, *P < 0.05. (**F**) Percentage of PVN CRHNs colabeled for nFos in mice treated as in (D). Individual dots indicate different animals. Column heights indicate means, and error bars indicate SEM. Unpaired *t* test, *P < 0.05. POMC neuron silencing caused a significant reduction in the activation of CRHNs by restraint. (**G**) Plasma ACTH concentration after mice expressing hM4Di in ARC POMC neurons were injected with vehicle or CNO and then exposed to predator odor (TMT) for 10 min. Column heights indicate means, and error bars indicate SEM. POMC neuron silencing had no effect on the ACTH response to predator odor. (**H** and **I**) Plasma ACTH concentration (H) and percentage of PVN *Crh*^+^ neurons activated (nFos^+^) after mice expressing hM4Di in ARC POMC neurons were injected with vehicle or CNO and then exposed to restraint only for 5 min. Column heights indicate means, and error bars indicate SEM. Unpaired *t* test, *P < 0.05. Silencing of POMC neurons also reduced the stress hormone response and percentage of activated CRHNs using a 5-min exposure to restraint.

We next used chemogenetic silencing to ask whether ARC POMC neurons are required for the stress hormone response to physical restraint or predator odor. We injected the ARC of POMC-Cre mice with AAV-DIO-hM4Di-mCherry (Fig. 6, A to C, and fig. S7, B and C) (*24*). hM4Di silences neurons when it binds to CNO. We then injected the mice with CNO or vehicle, exposed them to physical restraint or the predator odor TMT, and measured plasma ACTH.

Silencing of ARC POMC neurons caused a marked reduction in the stress hormone response to physical restraint (Fig. 6D). Following infection of POMC neurons with the AAV encoding hM4Di, restraint-exposed mice injected with CNO showed a 66 ± 6% decrease in plasma ACTH compared with mice injected with vehicle. Conversely, mice infected with the control AAV and exposed to restraint showed similar levels of ACTH following injection of CNO or vehicle (Fig. 6E). Silencing of POMC neurons also produced a decrease of 40 ± 4% in CRHNs labeled for nFos following restraint, suggesting that the effect on ACTH levels was mediated by CRHNs (Fig. 6F).

In contrast, silencing of ARC POMC neurons had no effect on the stress hormone response to the predator odor TMT (Fig. 6G). Animals exposed to restraint for 5 min, instead of 10 min, showed a lower level of plasma ACTH more similar to that seen in response to TMT. However, silencing of POMC neurons in those animals also reduced the stress hormone response to restraint (by 37 ± 4%) (Fig. 6H). POMC silencing also caused a 44 ± 6% decrease in the percentage of nFos^+^ PVN CRHNs observed after restraint for 5 min (Fig. 6I). These results exclude the possibility that the silencing could reduce larger, but not smaller, stressor-induced increases in stress hormone.

Together, these results indicate that POMC neurons in the arcuate nucleus play an important part in the full stress hormone response to one psychological stressor, physical restraint, but not another, the predator odor TMT. ARC POMC neurons have previously been implicated in appetite suppression and satiety (*8*, *9*). One intriguing question is whether there are different subsets of POMC neurons that mediate their effects on appetite versus stress or whether the same neurons can affect both functions.

## DISCUSSION

Danger triggers a surge in blood stress hormones controlled by hypothalamic CRHNs, but how signals from diverse stressors navigate the complex neural circuits upstream of CRHNs is largely unknown. Here, we sought to identify molecular markers that could distinguish neurons upstream of CRHNs in different brain areas and thus allow interrogation of their functions. We first used single-cell transcriptomics to identify CRHN receptors for neurotransmitters and neuromodulators and then viral tracing to pinpoint the locations of upstream neurons expressing ligands of those receptors. Glutamatergic and GABAergic upstream neurons were found in multiple brain areas. However, neuropeptide ligands were confined to upstream neurons in selected brain areas, providing molecular markers for specific subsets of upstream neurons. Combining functional and viral tracing approaches, we identified brain areas with upstream neurons activated by individual stressors, providing additional information about the functional organization of the upstream circuits. Further analysis showed that POMC neurons in ARC can be activated by restraint stress. Like restraint, chemogenetic activation of POMC neurons induced a stress hormone response. Moreover, silencing POMC neurons markedly reduced the stress hormone response to restraint but had no effect on the response to a predator odor. These results identify a molecularly defined subset of neurons that plays a key and selective role in the stress hormone response to a specific stressor.

The present studies indicate that CRHNs express a large number and variety of receptors for neurotransmitters and neuromodulators and can therefore be regulated by a varied array of signaling molecules released from upstream neurons (Fig. 1). All CRHNs expressed ionotropic receptors for both glutamate and GABA, suggesting that they can all be rapidly activated or inhibited by upstream glutamatergic or GABAergic neurons. In contrast, GPCRs for neuromodulators were typically expressed in subsets of CRHNs, suggesting that the excitability of different CRHNs may be modulated by different constellations of neuromodulatory inputs. Thus, each CRHN may be poised to adjust its activity and thereby influence stress hormone levels, according to a relatively unique combination of inputs from upstream neurons.

These studies identify molecular markers for subsets of neurons upstream of CRHNs in numerous brain areas (Fig. 2). These markers help to generate a combined molecular-anatomical map of the components of CRHN upstream circuits. They also provide molecular tools that can be used in future studies to investigate the functions of subsets of upstream neurons in defined areas. Most upstream areas contained some upstream neurons that were glutamatergic and some that were GABAergic. Glutamatergic inputs to CRHNs might convey excitatory signals about different stressors to CRHNs, whereas GABAergic inputs might be involved in tonic inhibition of CRHNs that could permit activation of CRHNs by some stressors via disinhibition (*3*, *5*, *13*, *14*). It is also conceivable that some GABAergic inputs permit blocking of stress hormone responses by particular stimuli, as reported for certain odors (*17*, *18*). In sharp contrast to glutamate and GABA, upstream neurons expressing neuropeptide ligands of CRHN receptors mapped to subsets of upstream neurons in specific areas, which differed for different neuropeptides. Together, these findings suggest that the activation state of CRHNs, and thereby stress hormone levels, is likely to be controlled by a combination of upstream signaling molecules, which could fine-tune the balance between excitatory, inhibitory, and neuromodulatory inputs to optimize CRHN function.

These analyses also provide information about neural circuits that convey signals about different stressors to CRHNs. Experiments using two different stressors, physical restraint and a predator odor, showed stressor activation of upstream neurons in specific brain areas (Fig. 3). Upstream neurons in the BNSTa were activated by either stressor, whereas those in ARC were activated only by restraint and those in LPGi only by predator odor. It cannot be excluded that additional upstream areas also play a role in responses to these stressors. Nonetheless, these studies indicate that information about a single stressor can reach CRHNs via different brain areas, and thus different parallel channels. They further indicate that one area can convey information to CRHNs about different stressors. Last, they suggest that some areas can convey signals about one specific stressor, but not another.

Experiments to localize neuropeptide ligands of CRHN receptors to upstream neurons showed that ~40% of upstream neurons in ARC express *Pomc* (Fig. 2J). Consistent with a previous finding that restraint activates ARC POMC neurons in rat (*23*), our experiments show activation of ARC POMC neurons upstream of CRHNs by restraint, although not a predator odor (Fig. 4). Chemogenetic experiments further indicate that activation of the POMC neurons can induce an increase in stress hormone, thus mimicking the response to a stressor (Fig. 5). Moreover, silencing POMC neurons markedly reduces the stress hormone response to restraint but has no effect on the stress hormone response to predator odor (Fig. 6). ARC POMC neurons have long been linked to satiety and appetite suppression (*8*, *9*). The present experiments indicate that they also play a major and selective role in the stress hormone response to a particular psychological stressor. Whether these two functions are mediated by separate subpopulations of ARC POMC neurons (*28*) is currently unknown.

## MATERIALS AND METHODS

### Mice

Mice aged 2 to 3 months were used. CRH-IRES-Cre (“CRH-Cre”) mice were generated previously (*10*). POMC-Cre mice (JAX, 005965), Ai14 mice with Cre-dependent expression of tdTomato (“Ai14-tdTomato reporter mice”) (JAX, 007914), and Ai6 mice with Cre-dependent expression of ZsGreen (“Ai6-ZsGreen reporter mice”) (JAX, 007906) were purchased from The Jackson Laboratory. All procedures involving mice were approved by the Fred Hutchinson Cancer Research Center Institutional Animal Care and Use Committee. Male and female mice were used in all experiments, with similar numbers where possible. No statistical methods were used to predetermine sample size. Animals were randomly chosen for experimental subjects. Animals were excluded from certain experiments. For chemogenetic activation or silencing of POMC neurons, the animals used had mCherry^+^ cells indicative of viral infection only in the ARC.

### Single-cell RNA-seq

Complementary DNA (cDNA) libraries were prepared from single CRHNs as previously described (*12*, *29*) with some modifications. Briefly, the PVN was isolated from CRH-Cre mice crossed with Ai14 mice, dissociated single cells were plated on coverslips, and single fluorescent cells were transferred to individual tubes using microcapillary pipettes (*12*). Using the Smart-Seq2 method (*29*), full-length cDNAs were prepared from mRNAs in each cell. Briefly, single cells were lysed and polyadenylated mRNA was reverse-transcribed by using a template switching method. cDNAs were then amplified using KAPA polymerase (Kapa Biosystems). After purification using AMPure XP beads (Beckman Coulter), cDNA libraries were prepared for sequencing using the Illumina TruSeq DNA Sample Prep Kit, as described previously (*12*). Briefly, cDNAs were fragmented to ~300 base pairs (bp), ligated to adaptors, and polymerase chain reaction (PCR)–amplified with adaptor primers.

Samples were subjected to multiplexed sequencing using an Illumina HiSeq 2500 instrument and a paired-end, 50-bp read-length sequencing strategy. Image analysis and base calling were conducted with Illumina Real-Time Analysis version 1.18 software, followed by demultiplexing of indexed reads and generation of FASTQ files with Illumina’s bcl2fastq Conversion Software (version 1.8.4). Low-quality reads were discarded before adapter trimming using Trim Galore (version 0.4.4; available at www.bioinformatics.babraham.ac.uk/projects/trim_galore/) with the options “-- adapter AAGCAGTGGTATCAACGCAGAGTAC --stringency 8 --quality 0 -e 0.15 --length 20 --paired --retain_unpaired”. The default options in GSNAP (GSNAP ref) (version 2014-12-29) (*30*) were used to align reads to the mouse genome (University of California Santa Cruz mm9 assembly and gene models). Gene-level counts were generated from GSNAP alignments using the Python package HTSeq (version 0.6.1) (HTSeq ref), using the “union” overlap mode (*31*).

We generated a list of receptor genes with Fragments Per Kilobase of transcripts per Million reads (FPKM) ≥1 in at least two cells. Lists of ligand-gated ion channels and GPCRs for neurotransmitters or neuromodulators were obtained from the International Union of Basic and Clinical Pharmacology/British Pharmacological Society website (www.guidetopharmacology.org). For previous sequencing data available in the Gene Expression Omnibus (GSE74672) [from Romanov *et al.* (*15*, *16*)], we included GPCRs for neurotransmitters/neuromodulators expressed in at least 2 of 86 neurons expressing *Crh* (≥1 molecule per cell) out of 898 hypothalamic neurons.

### Viral vectors

#### PRV

PRVB177 was propagated following methods described previously (*7*). Briefly, to propagate PRV177, PK15 cells (American Type Culture Collection) were infected with the virus using a multiplicity of infection of 0.1 to 0.01. After infection, cells showed a prominent cytopathic effect (~2 days). They were harvested by scraping, and the cell material was frozen using liquid nitrogen and then quickly thawed in a 37°C water bath. After three freeze-thaw cycles, cell debris was removed by centrifugation twice at 1000*g* for 5 min, and the supernatant was then used for experiments. The titer of viral stocks was determined using standard plaque assays on PK15 cells (*32*), with titers expressed in plaque-forming units (pfu).

#### AAVs

Serotype 8 AAVs were used with Cre recombinase–dependent flexstop cassettes that permit expression of mCherry-fused hM3Dq, mCherry-fused hM4Di, or mCherry under the control of the human synapsin promoter (AAV-DIO-hM3Dq-mCherry, AAV-DIO-hM4Di-mCherry, and AAV-DIO-mCherry) (*24*). The viruses were purchased from the Vector Core at the University of North Carolina at Chapel Hill. Amount used is described in virus particles (vp).

### Stereotaxic injection

Viruses were injected into the brain using a Stereotaxic Alignment System (David Kopf Instruments) with an inhalation anesthesia of 2.5% isoflurane. Virus suspensions [PRVs, 1 × 10^6^ to 1.5 × 10^6^ pfu (1 μl); AAVs, 1 × 10^9^ to 3 × 10^9^ vp (330 nl)] were loaded into a 1-μl syringe and injected at 100 nl/min. The needle was inserted to the target locations on the basis of a stereotaxic atlas [anterior-posterior (AP), −0.4 mm; medial-lateral (ML), ±0.3 mm; dorsal-ventral (DV), −5.0 mm for PVN; AP, −1.5 mm; ML, ±0.2 mm; DV, −5.9 mm for ARC]. After recovery, animals were singly housed with regular 12-hour dark/12-hour light cycles, and food and water were provided ad libitum.

### Stressors

#### Restraint stress

Mice were placed individually in a restrainer (a transparent plastic cylinder) (*21*) located in their home cage. The restrainer had a hole in the end near the tip of the animal’s nose. A piece of filter paper (1.5 cm by 2 cm) with absorbed 50 μl of distilled water was placed near the hole in the cylinder.

#### Predator odor stress

Mice were exposed to a predator odor or distilled water as described previously (*33*). A piece of filter paper [impregnated with 50 μl of 85 mM TMT (Contech) diluted in water or water alone] was dropped gently into one end of the home cage.

For detection of nFos in PRV-infected cells, CRH-Cre mice injected with PRVB177 3 days earlier were exposed to stressors or water for 5 min. In chemogenetic assays, mice injected with AAVs (AAV-DIO-hM4Di-mCherry) 2 weeks earlier were exposed to stressors for 10 min or 5 min. All stressors and control exposures were given between 10:00 a.m. and 12:00 p.m.

### Plasma ACTH and corticosterone assays

Plasma ACTH assays were performed as described previously (*7*). Briefly, after mice were euthanized by cervical dislocation and decapitation, trunk blood was collected directly into blood collection tubes (Becton Dickinson) containing 50 μl of aprotinin (Phoenix Pharmaceuticals). Plasma was obtained by centrifugation at 1600*g* for 15 min at 4°C and stored at −80°C. Plasma ACTH concentrations were measured using the ACTH ELISA Kit (MD Biosciences), according to the manufacturer’s instructions, with the following modifications: (i) 100 μl of the controls or blood plasma combined with 100 μl of PBS (phosphate-buffered saline; pH 7.4) was used in place of 200 μl of plasma, and (ii) the results were assessed with the QuantaRed Enhanced Chemifluorescent HRP Substrate (Thermo Fisher Scientific). Plasma corticosterone concentrations were measured using the Corticosterone ELISA Kit (Abcam), according to the manufacturer’s instructions, with the following modifications: (i) Plasma was diluted 25 times, instead of 100 times, with buffer M, and (ii) the results were assessed with the QuantaRed Enhanced Chemifluorescent HRP Substrate. Fluorescence was measured with a CytoFluor 4000 plate reader (Applied Biosystems).

### In situ hybridization and immunofluorescence

In situ hybridization was performed essentially as described previously (*7*, *12*), with some experiments using additional steps for triple staining. Coding region fragments of *Adcyap1r1*, *Sstr1*, *Calcr*, *Galr2*, *Npffr1*, *Cnr1*, *Mc3r*, *Drd2*, *Mchr1*, *Oprk1*, *Vglut1*, *Vglut2*, *Gad1*, *Gad2*, *Cgrp*, *Gal*, *Adcyap1*, *Penk*, *Pomc*, *Sst*, *and c-Fos* and the first intron sequence of *c-Fos* mRNA (for nFos staining) were isolated from mouse brain cDNA or mouse genomic DNA using PCR and cloned into the pCR4 TOPO vector (Thermo Fisher Scientific). Digoxigenin (DIG)–, dinitrophenol (DNP)–, or fluorescein (FLU)–labeled complementary RNA (cRNA) probes (riboprobes) were prepared using the DIG or FLU RNA Labeling Mix (Roche) or DNP RNA labeling mix containing DNP-11-UTP (NEL555001EA, PerkinElmer) and nucleoside triphosphates (Roche). CRH-Cre crossed with Ai6 reporter mice were perfused transcardially with 4% paraformaldehyde (PFA). Their brains were then soaked at 4°C in 4% PFA for 4 hours and in 30% sucrose for 48 hours, then frozen in OCT (Sakura), and cut into 14-μm coronal sections using a cryostat. Brains of CRH-Cre mice infected 3 days earlier with PRVB177, or wild-type animals, were fresh-frozen in OCT and cut into 20-μm coronal sections using a cryostat. Brain sections were hybridized to DIG-, DNP-, and/or FLU-labeled cRNA probes at 56°C for 13 to 16 hours.

#### Costaining with GFP antibodies to detect CRHNs and with GPCR riboprobes

Sections from Ai6;CRH-Cre mice were hybridized with DIG-labeled GPCR riboprobes. After hybridization, sections were washed twice in 0.2× SSC at 63°C for 30 min and incubated at 37°C for 2 hours with peroxidase (POD)–conjugated anti-DIG antibodies (Roche, #11207733910; 1:2000) and goat anti-GFP antibodies to detect the Ai6 reporter ZsGreen (Rockland, #600-101-215; 1:1000). Sections were then washed three times for 5 min at room temperature (RT) in TNT [0.1 M tris-HCl (pH7.5), 0.5 M NaCl, and 0.05% Tween] buffer and then treated using the TSA Plus Cy3 Kit (PerkinElmer). Sections were then washed three times for 5 min at RT in TNT buffer and incubated with DAPI (4′,6-diamidino-2-phenylindole) (0.5 μg ml^−1^) and Alexa Fluor 488 donkey anti-goat immunoglobulin G (IgG) (Thermo Fisher Scientific, #A11055; 1:1000) at RT for 1 hour and washed. Sections were coverslipped with Fluoromount-G (Southern Biotech).

#### Costaining for HA (PRVB177) and riboprobes for expression of ligands or nFos mRNA

On d3pi of CRHNs with PRVB177, brain sections were hybridized with DIG-labeled riboprobes for CRHN receptor ligands or *c-Fos* and nFos. After hybridization, sections were washed twice in 0.2× SSC at 63°C for 30 min and incubated with POD-conjugated anti-DIG antibodies (Roche, #11207733910; 1:2000) and biotinylated anti-HA antibodies (BioLegend, #901505; 1:300) at 37°C for 2 hours. Sections were then washed three times for 5 min at RT in TNT buffer and then treated using the TSA Plus FLU Kit (PerkinElmer). Sections were then washed three times for 5 min at RT in TNT buffer and incubated with DAPI (0.5 μg ml^−1^) and Alexa Fluor 555–streptavidin (Thermo Fisher Scientific, #32355; 1:1000) at RT for 1 hour and washed. Sections were coverslipped with Fluoromount-G (Southern Biotech).

#### Costaining for HA (PRVB177), nFos mRNA, and Pomc mRNA

On d3pi of CRHNs with PRVB177, sections were hybridized with DIG-labeled riboprobes for nFos and *c-Fos* and a DNP-labeled *Pomc* riboprobe. After hybridization, sections were washed twice in 0.2× SSC at 63°C for 30 min and incubated with POD-conjugated anti-DIG antibodies (Roche, #11207733910; 1:2000), rabbit anti–DNP-KLH antibodies (Molecular Probes, #A6430; 1:200), and biotinylated anti-HA antibodies (BioLegend, #901505; 1:300) at 37°C for 2 hours. Sections were then washed three times for 5 min at RT in TNT buffer and treated using the TSA Plus FLU Kit (PerkinElmer). Sections were then washed three times for 5 min at RT in TNT buffer and incubated with DAPI (0.5 μg ml^−1^), Alexa Fluor 555–streptavidin (Thermo Fisher Scientific, #32355; 1:1000), and Alexa Fluor 647 donkey anti-rabbit IgG (Thermo Fisher Scientific, #A21447; 1:1000) and at RT for 1 hour and washed. Sections were coverslipped with Fluoromount-G.

#### Costaining for Crh mRNA and nFos mRNA

After hybridization, sections were washed twice in 0.2× SSC at 63°C for 30 min, blocked with the Streptavidin/Biotin Blocking Kit (Vector Laboratories), incubated with POD-conjugated anti-FLU antibodies (Roche, #11426346910; 1:300) and alkaline phosphatase–conjugated anti-DIG antibodies (Roche, #11093274910; 1:300) at RT for 2 hours, and then treated using the TSA Plus Biotin Kit (PerkinElmer). Sections were next incubated with DAPI (0.5 μg ml^−1^) and Alexa Fluor 488–streptavidin (Thermo Fisher Scientific; 1:1000) at RT for 30 min, incubated with HNPP Fluorescent Detection Set (Roche) at RT for 1 hour, and then coverslipped with Fluoromount-G.

### Chemogenetic activation and silencing

Chemogenetic experiments were performed as described previously (*7*) with some modifications.

#### Activation

AAV-DIO-hM3Dq-mCherry was injected into the ARC of POMC-Cre mice by stereotaxic injection (see above). At 2 weeks after injection, mice were intraperitoneally injected with CNO (Sigma) (5.0 mg kg^−1^ body weight) or vehicle (0.4% dimethyl sulfoxide dissolved in saline). Thirty minutes later, trunk blood and brain were collected, and the blood was used for plasma ACTH assays (see above). Brains were fixed by soaking in 4% PFA in PBS for 4 hours, soaked in 30% sucrose for 24 hours, frozen in OCT, and cut into 20-μm coronal sections using a cryostat. Brain sections were washed twice with PBS, permeabilized with 0.5% Triton X-100 in PBS for 5 min, washed twice with PBS, blocked with TNB [0.1 M tris-HCl (pH 7.5), 0.5 M NaCl, and 0.5% Blocking Reagent] (PerkinElmer) for 1 hour at RT, and then incubated with rabbit anti-RFP (red fluorescent protein) (to detect mCherry) (Rockland, #600-410-379; 1:500) and goat anti-POMC (Phoenix Pharmaceuticals, #H-029-30; 1:500) diluted in TNB at 4°C overnight. Sections were then washed three times with TNT, incubated with Alexa Fluor 555 donkey anti-rabbit IgG (Thermo Fisher Scientific, #A31572; 1:1000) and DAPI (0.5 μg ml^−1^) for 1 hour at RT, and washed three times with TNT. Slides were coverslipped with Fluoromount-G (Southern Biotech).

#### Silencing

AAV-DIO-hM4Di-mCherry was injected into the ARC of POMC-Cre mice by stereotaxic injection (see above). At 2 weeks after injection, mice were intraperitoneally injected with CNO (5.0 mg kg^−1^ body weight) or vehicle. Thirty minutes later, mice were exposed to water, restraint stress, or a predator odor (TMT) for 10 min (or 5 min) (see above). Trunk blood and brain were then collected. Blood was used for plasma ACTH assays, and brains were treated and immunostained with rabbit anti-RFP and goat anti-POMC antibodies (see above).

### Cell counting

Cell counting was performed as described previously (*7*). Images were collected using an AxioCam camera and AxioImager.Z2 microscope with an apotome device (Zeiss). Images were acquired with auto-exposure setting, because background between different slices and animals is different. No additional postprocessing was performed on any of the collected images for counting. Counting was conducted blindly. Brain structures were identified microscopically and in digital photos using a mouse brain atlas (*34*). Every fifth section was analyzed for all experiments. For the data shown in Figs. 3 and 4, cells were counted in a given area in only one hemisphere, the hemisphere that contained the most PRV^+^ cells. For costaining experiments for PRV and ligands of receptors in CRHNs shown in Fig. 2, brain areas containing colabeled cells for a given ligand (at least 10 colabeled cells in a given area) in more than two animals were only included. The percentage of PRV^+^ cells with nFos labeling among all PRV^+^ cells lacking cytoplasmic *Fos* labeling was calculated.

### Statistical analysis

All data are shown as means ± SEM. Data were tested with the Shapiro-Wilk test for normality. For data with a normal distribution, the unpaired *t* test was used to compare two groups to analyze statistical significance. For data without a normal distribution, the Mann-Whitney *U* test or the Kruskal-Wallis test with post hoc Dunn’s test was used to compare two groups or more than two groups, respectively. All tests were two-sided.

### Abbreviations for brain areas

Abbreviations used for brain areas are according to our previous report (*7*): AH, anterior hypothalamic area; ARC, arcuate hypothalamic nucleus; AVPe, anteroventral periventricular nucleus; BNSTa, bed nucleus of the stria terminalis, anterior part; BNSTp, bed nucleus of the stria terminalis, posterior part; DMH, dorsomedial hypothalamic nucleus; LH, lateral hypothalamic area; LPAG, lateral periaqueductal gray; LPGi, lateral paragigantocellular nucleus; LPO, lateral preoptic area; LS, lateral septal nucleus; MnPO, median preoptic nucleus; MPA, medial preoptic area; MPO, medial preoptic nucleus; MTu, medial tuberal nucleus; NTS, nucleus of the solitary tract; PBN, parabrachial nucleus; Pe, periventricular nucleus of the hypothalamus; PH, posterior hypothalamic nucleus; PLH, peduncular part of lateral hypothalamus; PMV, premammillary nucleus, ventral part; SCh, suprachiasmatic nucleus; SHy, septohippocampal nucleus; StHy, striohypothalamic nucleus; VMH, ventromedial hypothalamic nucleus; ZI, zona incerta.

## Supplementary Material

aay5366_SM.pdf

## SUPPLEMENTARY MATERIALS

Supplementary material for this article is available at http://advances.sciencemag.org/cgi/content/full/6/12/eaay5366/DC1 Fig. S1. Strategy to identify molecular markers for neurons upstream of CRHNs. Fig. S2. Individual neurotransmitter and biogenic amine receptors expressed by CRHNs. Fig. S3. In vivo verification of receptor expression in CRHNs. Fig. S4. Ligands of CRHN receptors in neurons upstream of CRHNs. Fig. S5. Numbers and percentages of PRV^+^, PRV^+^nFos^+^, and PRV^+^Pomc^+^nFos^+^ neurons. Fig. S6. POMC neurons do not affect AVP neurons in PVN. Fig. S7. Locations of infected AAV-infected neurons.

## Appendix

View/request a protocol for this paper from *Bio-protocol*.

## References

[1] Makara G. B., Stark E., Kárteszi M., Palkovits M., Rappay G., Effects of paraventricular lesions on stimulated ACTH release and CRF in stalk-median eminence of the rat. Am. J. Physiol. 240, E441–E446 (1981). 6261587 10.1152/ajpendo.1981.240.4.E441

[2] Muglia L., Jacobson L., Majzoub J. A., Production of corticotropin-releasing hormone-deficient mice by targeted mutation in embryonic stem cells. Ann. N. Y. Acad. Sci. 780, 49–59 (1996). 8602739 10.1111/j.1749-6632.1996.tb15111.x

[3] Ulrich-Lai Y. M., Herman J. P., Neural regulation of endocrine and autonomic stress responses. Nat. Rev. Neurosci. 10, 397–409 (2009). 19469025 10.1038/nrn2647 PMC4240627

[4] Gillespie C. F., Phifer J., Bradley B., Ressler K. J., Risk and resilience: Genetic and environmental influences on development of the stress response. Depress. Anxiety 26, 984–992 (2009). 19750552 10.1002/da.20605 PMC2852579

[5] Pacák K., Palkovits M., Stressor specificity of central neuroendocrine responses: Implications for stress-related disorders. Endocr. Rev. 22, 502–548 (2001). 11493581 10.1210/edrv.22.4.0436

[6] Jaggi A. S., Bhatia N., Kumar N., Singh N., Anand P., Dhawan R., A review on animal models for screening potential anti-stress agents. Neurol. Sci. 32, 993–1005 (2011). 21927881 10.1007/s10072-011-0770-6

[7] Kondoh K., Lu Z., Lu Z., Ye X., Olson D. P., Lowell B. B., Buck L. B., A specific area of olfactory cortex involved in stress hormone responses to predator odours. Nature 532, 103–106 (2016). 27001694 10.1038/nature17156 PMC5094457

[8] Andermann M. L., Lowell B. B., Toward a wiring diagram understanding of appetite control. Neuron 95, 757–778 (2017). 28817798 10.1016/j.neuron.2017.06.014 PMC5657399

[9] Sternson S. M., Eiselt A.-K., Three pillars for the neural control of appetite. Annu. Rev. Physiol. 79, 401–423 (2017). 27912679 10.1146/annurev-physiol-021115-104948

[10] Krashes M. J., Shah B. P., Madara J. C., Olson D. P., Strochlic D. E., Garfield A. S., Vong L., Pei H., Watabe-Uchida M., Uchida N., Liberles S. D., Lowell B. B., An excitatory paraventricular nucleus to AgRP neuron circuit that drives hunger. Nature 507, 238–242 (2014). 24487620 10.1038/nature12956 PMC3955843

[11] Madisen L., Zwingman T. A., Sunkin S. M., Oh S. W., Zariwala H. A., Gu H., Ng L. L., Palmiter R. D., Hawrylycz M. J., Jones A. R., Lein E. S., Zeng H., A robust and high-throughput Cre reporting and characterization system for the whole mouse brain. Nat. Neurosci. 13, 133–140 (2010). 20023653 10.1038/nn.2467 PMC2840225

[12] Hanchate N. K., Kondoh K., Lu Z., Kuang D., Ye X., Qiu X., Pachter L., Trapnell C., Buck L. B., Single-cell transcriptomics reveals receptor transformations during olfactory neurogenesis. Science 350, 1251–1255 (2015). 26541607 10.1126/science.aad2456 PMC5642900

[13] Aguilera G., Liu Y., The molecular physiology of CRH neurons. Front. Neuroendocrinol. 33, 67–84 (2012). 21871477 10.1016/j.yfrne.2011.08.002 PMC4341841

[14] Levy B. H., Tasker J. G., Synaptic regulation of the hypothalamic-pituitary-adrenal axis and its modulation by glucocorticoids and stress. Front. Cell. Neurosci. 6, 24 (2012). 22593735 10.3389/fncel.2012.00024 PMC3349941

[15] Romanov R. A., Zeisel A., Bakker J., Girach F., Hellysaz A., Tomer R., Alpár A., Mulder J., Clotman F., Keimpema E., Hsueh B., Crow A. K., Martens H., Schwindling C., Calvigioni D., Bains J. S., Máté Z., Szabó G., Yanagawa Y., Zhang M.-D., Rendeiro A., Farlik M., Uhlén M., Wulff P., Bock C., Broberger C., Deisseroth K., Hökfelt T., Linnarsson S., Horvath T. L., Harkany T., Molecular interrogation of hypothalamic organization reveals distinct dopamine neuronal subtypes. Nat. Neurosci. 20, 176–188 (2017). 27991900 10.1038/nn.4462 PMC7615022

[16] Romanov R. A., Alpár A., Hökfelt T., Harkany T., Molecular diversity of corticotropin-releasing hormone mRNA-containing neurons in the hypothalamus. J. Endocrinol. 232, R161–R172 (2017). 28057867 10.1530/JOE-16-0256

[17] Murakami T., Matsukawa M., Katsuyama N., Imada M., Aizawa S., Sato T., Stress-related activities induced by predator odor may become indistinguishable by hinokitiol odor. Neuroreport 23, 1071–1076 (2012). 23128452 10.1097/WNR.0b013e32835b373b

[18] Matsukawa M., Imada M., Murakami T., Aizawa S., Sato T., Rose odor can innately counteract predator odor. Brain Res. 1381, 117–123 (2011). 21266167 10.1016/j.brainres.2011.01.053

[19] Kobayakawa K., Kobayakawa R., Matsumoto H., Oka Y., Imai T., Ikawa M., Okabe M., Ikeda T., Itohara S., Kikusui T., Mori K., Sakano H., Innate versus learned odour processing in the mouse olfactory bulb. Nature 450, 503–508 (2017). 10.1038/nature06281 17989651

[20] E. Vernet-Maury, Trimethyl-thiazoline in fox feces: A natural alarming substance for the rat, in *Olfaction Taste*, H. Van der Starre, Ed. (IRL Press, 1980), vol. 407.

[21] Lee E. J., Son G. H., Chung S., Lee S., Kim J., Choi S., Kim K., Impairment of fear memory consolidation in maternally stressed male mouse offspring: Evidence for nongenomic glucocorticoid action on the amygdala. J. Neurosci. 31, 7131–7140 (2011). 21562275 10.1523/JNEUROSCI.4692-10.2011 PMC6703196

[22] Lin D., Boyle M. P., Dollar P., Lee H., Lein E. S., Perona P., Anderson D. J., Functional identification of an aggression locus in the mouse hypothalamus. Nature 470, 221–226 (2011). 21307935 10.1038/nature09736 PMC3075820

[23] Liu J., Garza J. C., Truong H. V., Henschel J., Zhang W., Lu X.-Y., The melanocortinergic pathway is rapidly recruited by emotional stress and contributes to stress-induced anorexia and anxiety-like behavior. Endocrinology 148, 5531–5540 (2007). 17673512 10.1210/en.2007-0745 PMC3708592

[24] Krashes M. J., Koda S., Ye C., Rogan S. C., Adams A. C., Cusher D. S., Maratos-Flier E., Roth B. L., Lowell B. B., Rapid, reversible activation of AgRP neurons drives feeding behavior in mice. J. Clin. Invest. 121, 1424–1428 (2011). 21364278 10.1172/JCI46229 PMC3069789

[25] Balthasar N., Coppari R., McMinn J., Liu S. M., Lee C. E., Tang V., Kenny C. D., McGovern R. A., Chua S. C. Jr., Elmquist J. K., Lowell B. B., Leptin receptor signaling in POMC neurons is required for normal body weight homeostasis. Neuron 42, 983–991 (2004). 15207242 10.1016/j.neuron.2004.06.004

[26] Armbruster B. N., Li X., Pausch M. H., Herlitze S., Roth B. L., Evolving the lock to fit the key to create a family of G protein-coupled receptors potently activated by an inert ligand. Proc. Natl. Acad. Sci. U.S.A. 104, 5163–5168 (2007). 17360345 10.1073/pnas.0700293104 PMC1829280

[27] Gomez J. L., Bonaventura J., Lesniak W., Mathews W. B., Sysa-Shah P., Rodriguez L. A., Ellis R. J., Richie C. T., Harvey B. K., Dannals R. F., Pomper M. G., Bonci A., Michaelides M., Chemogenetics revealed: DREADD occupancy and activation via converted clozapine. Science 357, 503–507 (2017). 28774929 10.1126/science.aan2475 PMC7309169

[28] Campbell J. N., Macosko E. Z., Fenselau H., Pers T. H., Lyubetskaya A., Tenen D., Goldman M., Verstegen A. M., Resch J. M., McCarroll S. A., Rosen E. D., Lowell B. B., Tsai L. T., A molecular census of arcuate hypothalamus and median eminence cell types. Nat. Neurosci. 20, 484–496 (2017). 28166221 10.1038/nn.4495 PMC5323293

[29] Picelli S., Faridani O. R., Björklund A. K., Winberg G., Sagasser S., Sandberg R., Full-length RNA-seq from single cells using Smart-seq2. Nat. Protocols 9, 171–181 (2014). 24385147 10.1038/nprot.2014.006

[30] Wu T. D., Nacu S., Fast and SNP-tolerant detection of complex variants and splicing in short reads. Bioinformatics 26, 873–881 (2010). 20147302 10.1093/bioinformatics/btq057 PMC2844994

[31] Anders S., Pyl P. T., Huber W., HTSeq—A Python framework to work with high-throughput sequencing data. Bioinformatics 31, 166–169 (2015). 25260700 10.1093/bioinformatics/btu638 PMC4287950

[32] Card J. P., Enquist L. W., Transneuronal circuit analysis with pseudorabies viruses. Curr. Protoc. Neurosci. 68, 1.5.1–1.5.39 (2001). 10.1002/0471142301.ns0105s68 PMC4165084 24984685

[33] Saraiva L. R., Kondoh K., Ye X., Yoon K.-H., Hernandez M., Buck L. B., Combinatorial effects of odorants on mouse behavior. Proc. Natl. Acad. Sci. U.S.A. 113, E3300–E3306 (2016). 27208093 10.1073/pnas.1605973113 PMC4988607

[34] K. Franklin, G. Paxino, *The Mouse Brain in Stereotaxic Coordinates* (Academic Press, ed. 3, 2008).

