# Supplementary material for: A psychological stressor conveyed by appetite-linked neurons
Source: Sci Adv. 2020 Mar 18;6(12):eaay5366. doi: 10.1126/sciadv.aay5366 (PMC7080447; doi:10.1126/sciadv.aay5366)
Supplement: aay5366_SM.pdf [file aay5366_SM.pdf]

Science Advances
AAAS

advances.sciencemag.org/cgi/content/full/6/12/eaay5366/DC1

Supplementary Materials for

A psychological stressor conveyed by appetite-linked neurons

Eun Jeong Lee, Naresh K. Hanchate, Kunio Kondoh, Ai Phuong S. Tong, Donghui Kuang, Andrew Spray, Xiaolan Ye, Linda B. Buck*

*Corresponding author. Email: lbuck@fhcrc.org

Published 18 March 2020, *Sci. Adv.* **6**, eaay5366 (2020)
DOI: 10.1126/sciadv.aay5366

This PDF file includes:

Fig. S1. Strategy to identify molecular markers for neurons upstream of CRHNs.
Fig. S2. Individual neurotransmitter and biogenic amine receptors expressed by CRHNs.
Fig. S3. In vivo verification of receptor expression in CRHNs.
Fig. S4. Ligands of CRHN receptors in neurons upstream of CRHNs.
Fig. S5. Numbers and percentages of PRV^+, PRV^+nFos^+, and $PRV^+Pomc^+nFos^+$ neurons.
Fig. S6. POMC neurons do not affect AVP neurons in PVN.
Fig. S7. Locations of infected AAV-infected neurons.

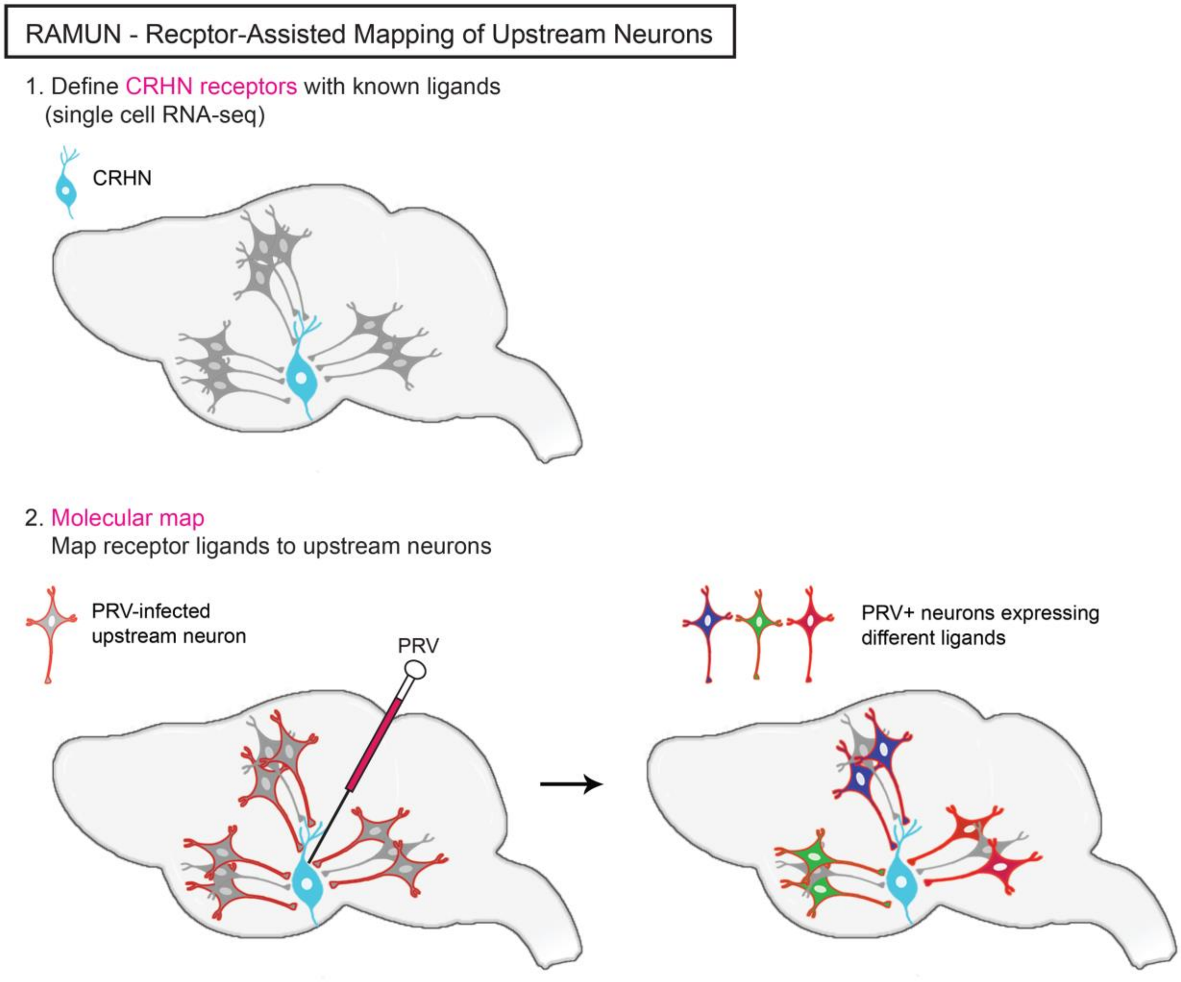

Fig. S1. Strategy to identify molecular markers for neurons upstream of CRHNs. The strategy employed was to first define CRHN receptors with known ligands using single cell RNA-seq (1) and then map receptor ligands to upstream neurons following PRV-infection of CRHNs (2). This strategy is termed “RAMUN” (receptor-assisted mapping of upstream neurons).

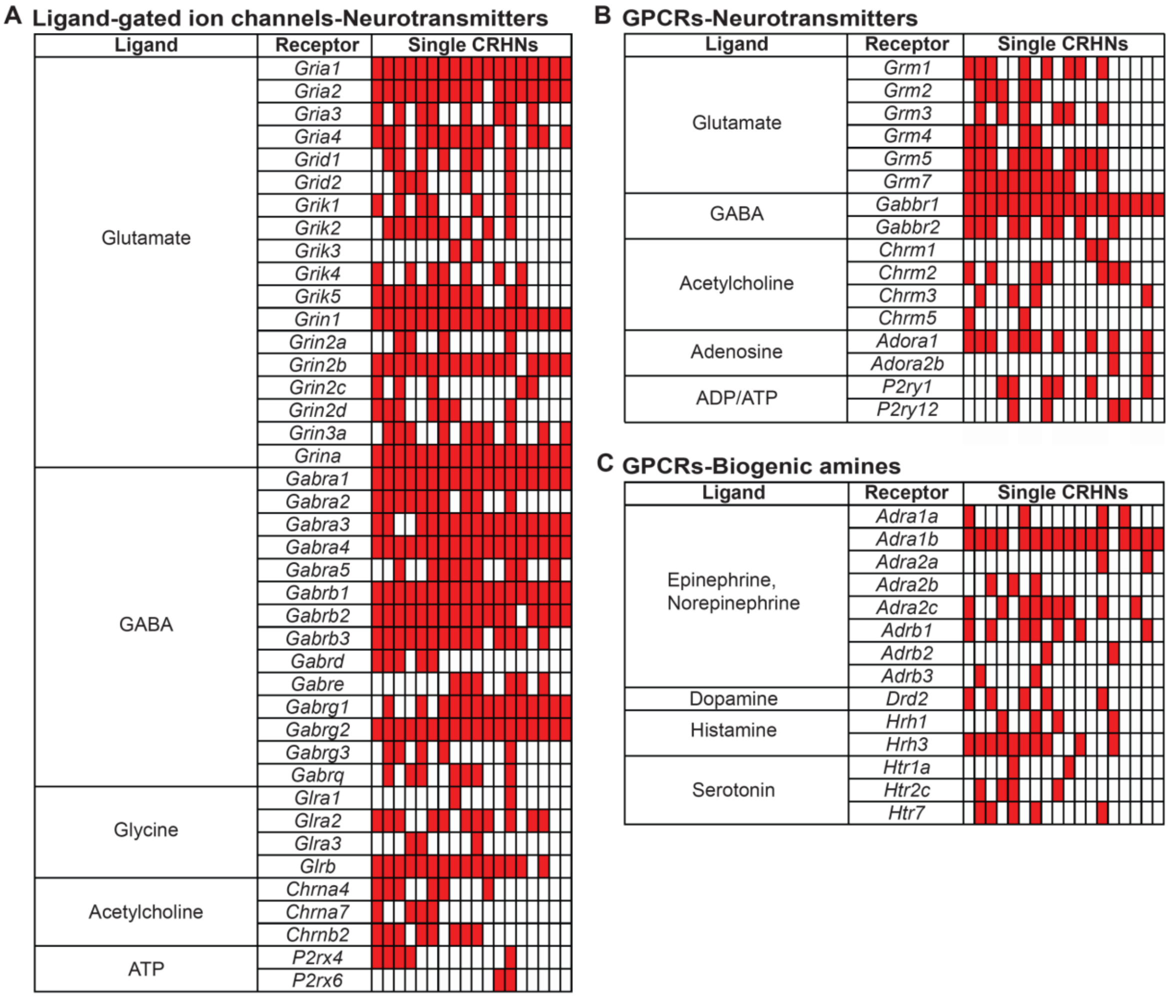

Fig. S2. Individual neurotransmitter and biogenic amine receptors expressed by CRHNs. Transcriptome analyses of single CRHNs showed expression of a variety of ligand-gated ion channels for neurotransmitters (**A**), GPCRs that recognize neurotransmitters (glutamate, GABA, glycine, acetylcholine, adenosine, or ADP/ATP) (**B**), and GPCRs that recognize biogenic amines (epinephrine, norepinephrine, dopamine, histamine, serotonin) (**C**). Boxes at right indicate individual CRHNs, with those expressing a receptor gene shown in red.

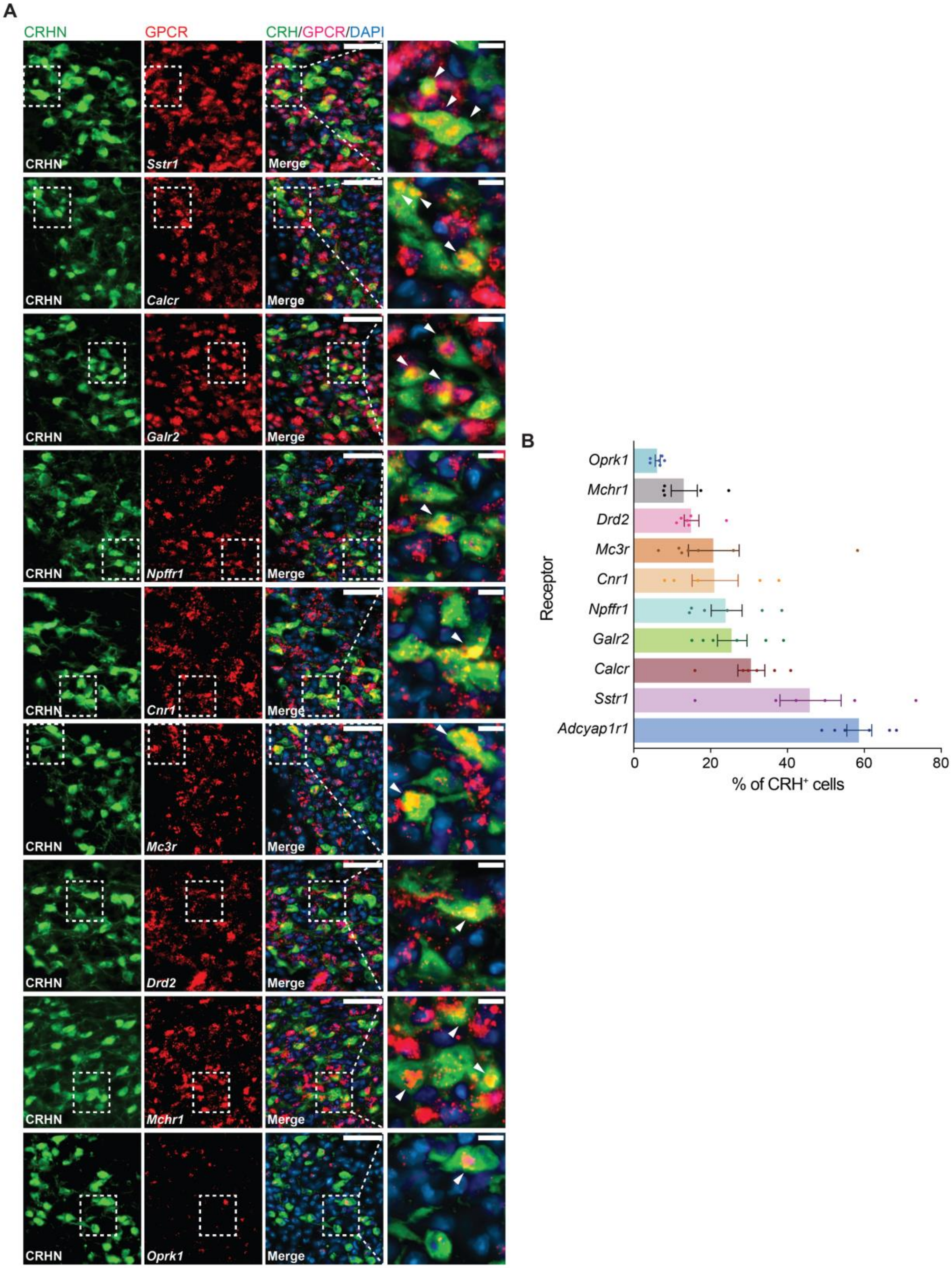
A
CRHN
GPCR
CRH/GPCR/DAPI
CRHN
Sstr1
Merge
Calcr
Galr2
Npffr1
Cnr1
Mc3r
Drd2
Mchr1
Oprk1
B
Receptor
Oprk1
Mchr1
Drd2
Mc3r
Cnr1
Npffr1
Galr2
Calcr
Sstr1
Adcyap1r1
0
20
40
60
80
% of CRH⁺ cells

Fig. S3. In vivo verification of receptor expression in CRHNs. (**A**) Photographs of PVN sections from CRH-Cre mice crossed with Ai6-ZsGreen reporter mice. Sections were costained with anti-GFP (ZsGreen) antibodies to identify CRHNs (green) and riboprobes for different GPCRs identified in CRHNs by transcriptome analyses (red) (GPCR). Sections were counterstained with DAPI (blue). Shown in different rows are experiments with riboprobes for *Adcyap1r1, Sstr1, Calcr, Galr2, Npffr1, Cnr1, Mc3r, Drd2, Mchr1,* and *Oprk1*. Higher magnifications of boxed areas showing CRHNs labeled for different receptors are shown on the right. Arrowheads indicate colabeled cells. Scale bars, 100 μm (left) and 20 μm (right). (**B**) The percentages of PVN CRH+ (ZsGreen+) neurons colabeled for different receptors varied, suggesting that there may be subsets of CRHNs with different sets of receptors. n=5-7 per receptor. Error bars indicate s.e.m.

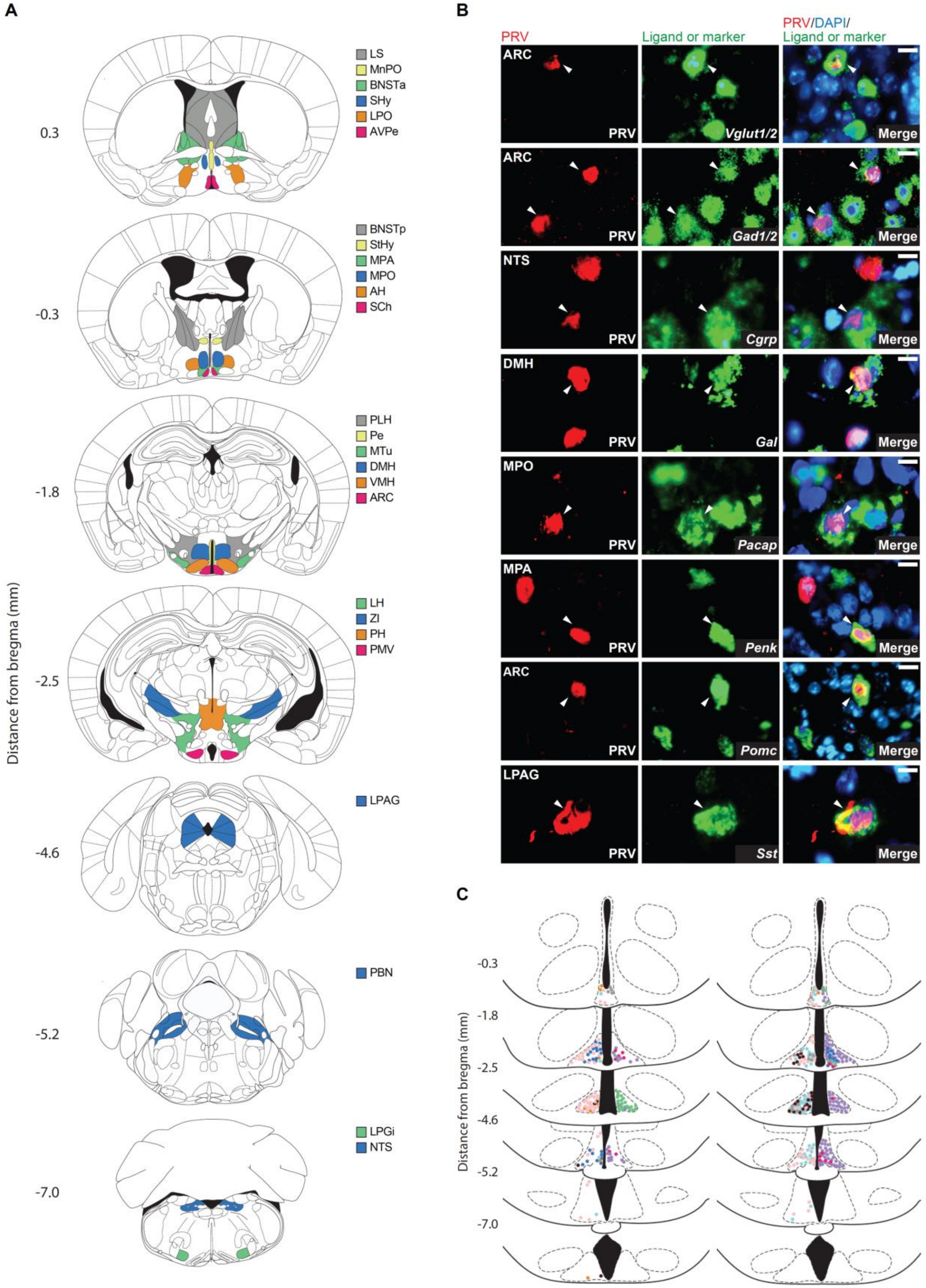

A
LS
MnPO
BNSTa
SHy
LPO
AVPe
0.3
BNSTp
StHy
MPA
MPO
AH
SCh
-0.3
PLH
Pe
MTu
DMH
VMH
ARC
-1.8
LH
ZI
PH
PMV
-2.5
LPAG
-4.6
PBN
-5.2
LPGi
NTS
-7.0
Distance from bregma (mm)
B
PRV
Ligand or marker
PRV/DAPI/
Ligand or marker
ARC
PRV
Vglut1/2
Merge
ARC
PRV
Gad1/2
Merge
NTS
PRV
Cgrp
Merge
DMH
PRV
Gal
Merge
MPO
PRV
Pacap
Merge
MPA
PRV
Penk
Merge
ARC
PRV
Pomc
Merge
LPAG
PRV
Sst
Merge
C
-0.3
-1.8
-2.5
-4.6
-5.2
-7.0
Distance from bregma (mm)

Fig. S4. Ligands of CRHN receptors in neurons upstream of CRHNs. (**A**) Diagrams showing brain areas containing PRV-infected neurons on d3pi. Twenty-six brain areas are indicated in different colors on coronal schematic brain sections along the anterior-posterior axis. Modified from Franklin and Paxinos, 2008 (*34*). (**B**) CRHNs were infected with PRVB177 and brain sections costained with anti-HA antibodies to label PRV+ neurons (red) (PRV) and riboprobes to detect expression of specific neurotransmitters or neuromodulators recognized by CRHN receptors (green) (Ligand or marker). Sections were counterstained with DAPI (blue). Each row shows images from one section, with the brain area indicated at left, the riboprobe indicated in the middle image, and a merged image shown at right. Arrowheads indicate PRV+ cells colabeled with riboprobes. Scale bar, 10 μm. (**C**) Two sets of schematic sections (left and right) show the locations of PRV+ *Pomc*+ neurons in 9 animals each (total of 18 animals). Dots indicate individual PRV+ *Pomc*+ cells and different colors indicate different animals. PRV+ *Pomc*+ cells from each animal are indicated in only one hemisphere.

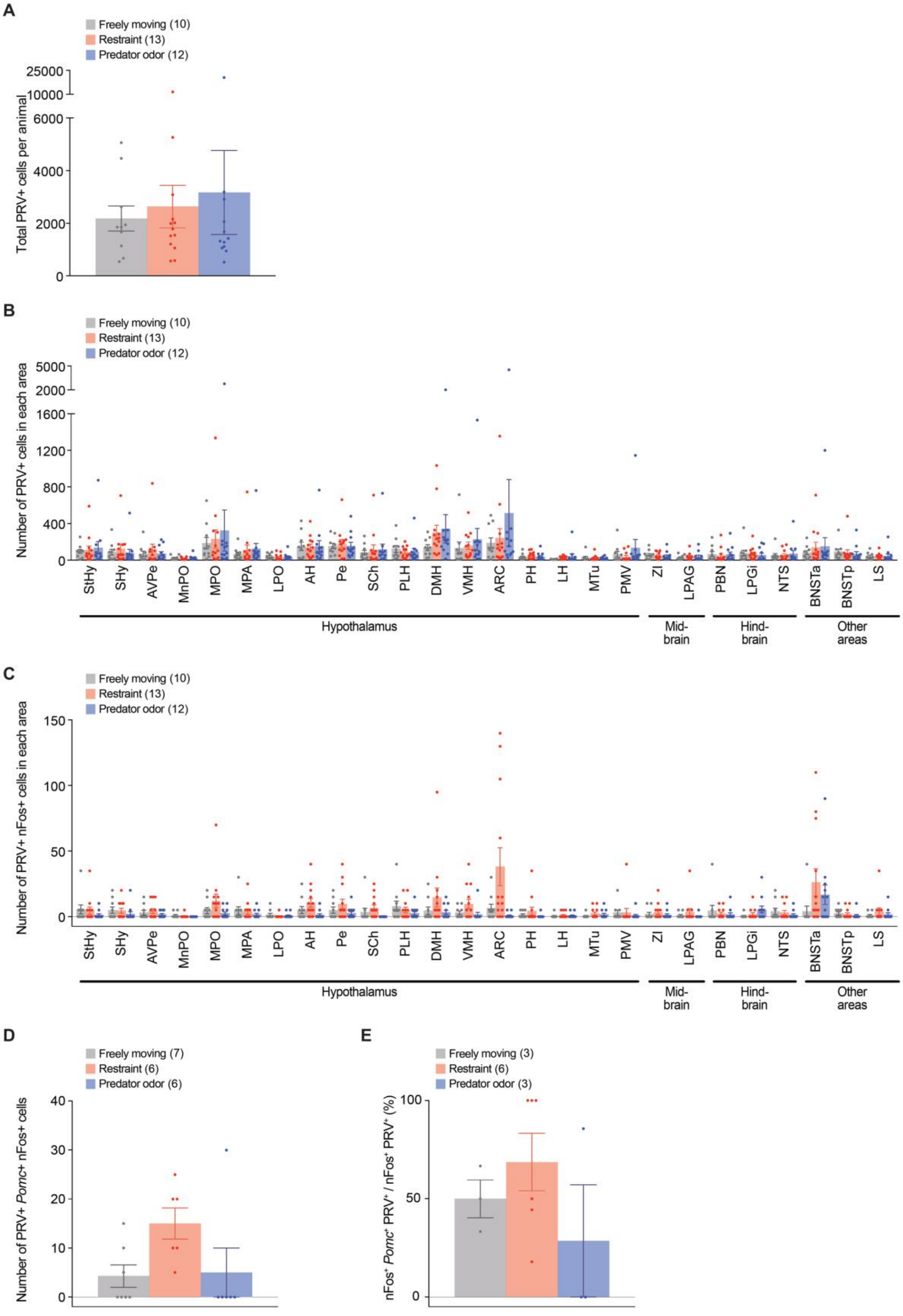
A
Freely moving (10)
Restraint (13)
Predator odor (12)
Total PRV+ cells per animal
25000
10000
6000
4000
2000
0
B
Freely moving (10)
Restraint (13)
Predator odor (12)
Number of PRV+ cells in each area
5000
2000
1600
1200
800
400
0
StHy
SHy
AVPe
MnPO
MPO
MPA
LPO
AH
Pe
SCh
PLH
DMH
VMH
ARC
PH
LH
MTu
PMV
ZI
LPAG
PBN
LPGi
NTS
BNSTa
BNSTp
LS
Hypothalamus
Mid-brain
Hind-brain
Other areas
C
Freely moving (10)
Restraint (13)
Predator odor (12)
Number of PRV+ nFos+ cells in each area
150
100
50
0
StHy
SHy
AVPe
MnPO
MPO
MPA
LPO
AH
Pe
SCh
PLH
DMH
VMH
ARC
PH
LH
MTu
PMV
ZI
LPAG
PBN
LPGi
NTS
BNSTa
BNSTp
LS
Hypothalamus
Mid-brain
Hind-brain
Other areas
D
Freely moving (7)
Restraint (6)
Predator odor (6)
Number of PRV+ Pomc+ nFos+ cells
40
30
20
10
0
E
Freely moving (3)
Restraint (6)
Predator odor (3)
nFos+ Pomc+ PRV+ / nFos+ PRV+ (%)
100
50
0

Fig. S5. Numbers and percentages of PRV$^+$, PRV$^+$nFos$^+$, and PRV$^+$Pomc$^+$nFos$^+$ neurons. (**A-C**) Total number of PRV+ neurons per animal (A) and number of PRV+ neurons (B) and number of PRV+nFos+ neurons (C) in each brain area shown in Fig. 3B. Numbers of animals are indicated in parentheses. Column heights indicate means and error bars indicate s.e.m.. Dots indicate values for individual animals. (**D**) Number of ARC PRV+ neurons in different brain areas that were *Pomc*+nFos+ and shown in Fig. 4C, D and fig. S5E. Numbers of animals are indicated in parentheses. Column heights indicate means and error bars indicate s.e.m.. Dots indicate values for individual animals. ARC PRV+ neurons with *Pomc* and nFos were seen in 3/7 freely moving animals, 6/6 animals exposed to restraint, and 1/6 animals exposed to predator odor. (**E**) Graph shows the percentage of ARC nFos+ PRV+ cells colabeled for *Pomc* in the experiments shown in Fig. 4C, D and fig. S5D. Numbers of animals are indicated in parentheses. Column heights indicate means and error bars indicate s.e.m.. Dots indicate values for individual animals. Animals exposed to restraint showed a higher proportion of colabeled cells than those exposed to predator odor or freely moving control animals. For animals in the Freely moving, Restraint, and Predator odor categories, respectively, 3/7, 6/6, and 3/6 contained ARC nFos+PRV+ neurons, and 3/7, 6/6, and 1/6 contained nFos+PRV+*Pomc*+ neurons. Results are shown for animals with ARC nFos+PRV+ neurons.

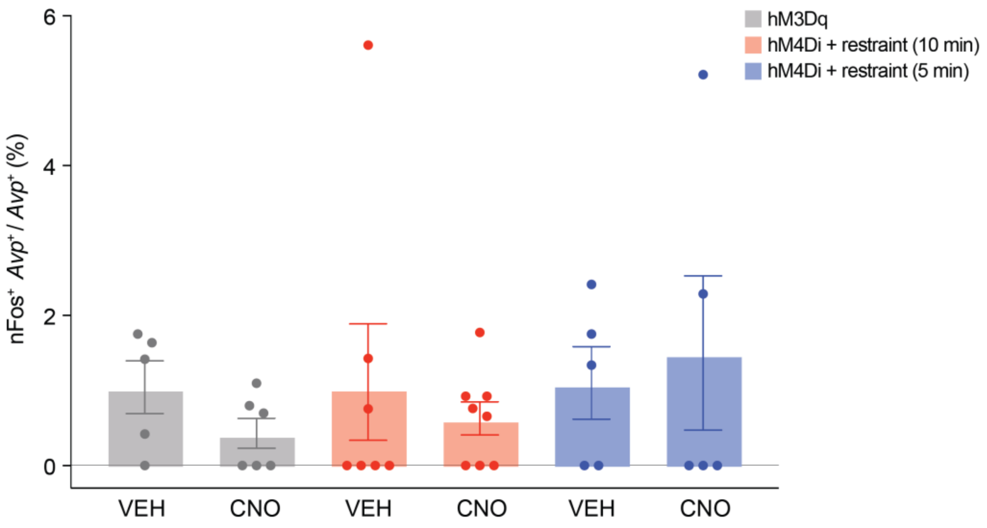

Fig. S6. POMC neurons do not affect AVP neurons in PVN. Percentage of PVN *Avp*+ neurons co-labelled for nFos in mice treated as in Fig. 5D, Fig. 6D and Fig. 6H. Individual dots indicate different animals. Column heights indicate means and error bars indicate s.e.m.. ARC POMC neurons were infected with an AAV encoding hM3Dq or hM4Di and animals injected with CNO or vehicle control (VEH). Animals infected with hM4Di were exposed to restraint for 10 min or 5 min. PVN sections were costained for nFos and *Avp*. Neither activation nor silencing of POMC neurons affected the percentage of *Avp*+ neurons labeled for nFos.

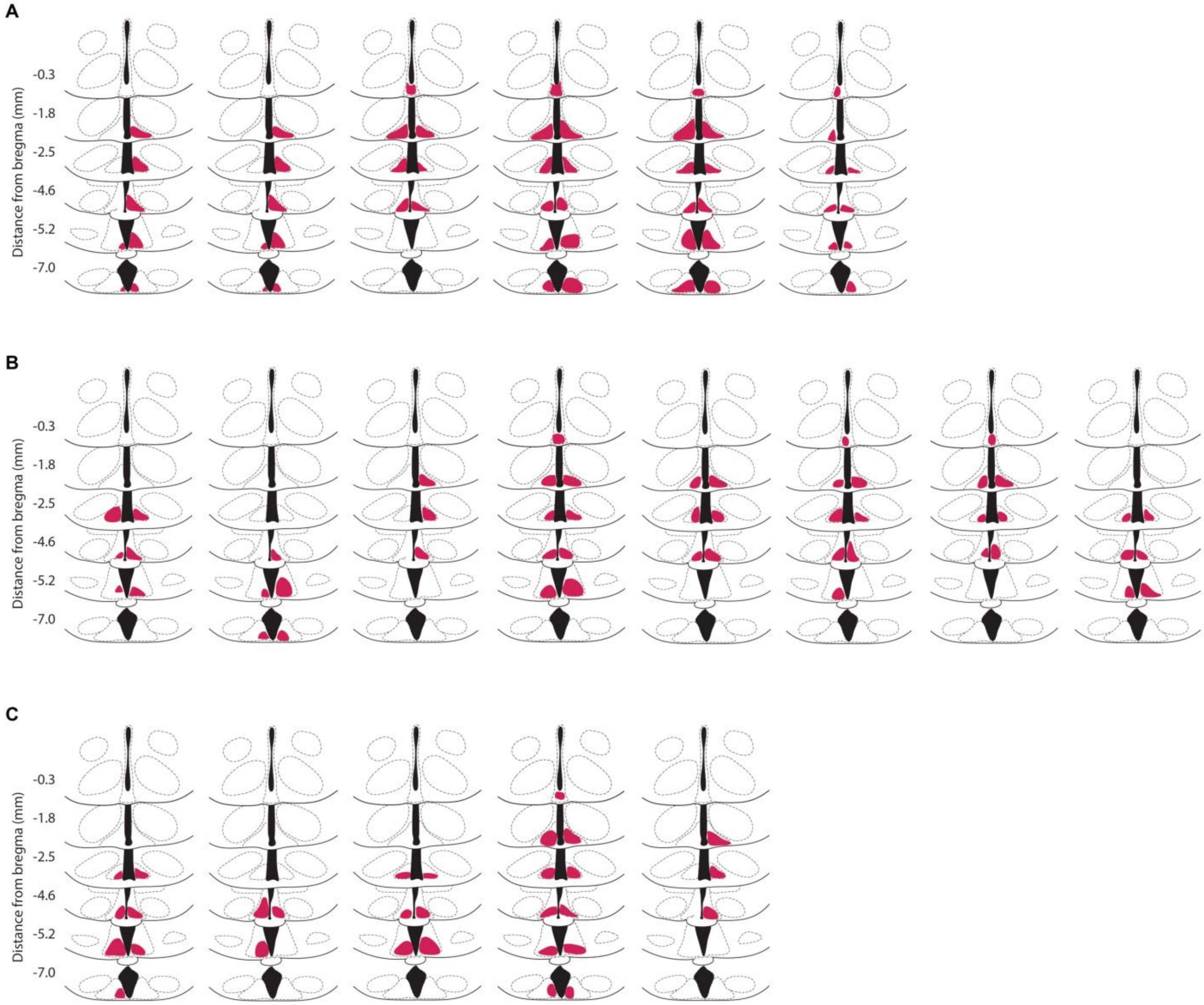

Fig. S7. Locations of infected AAV-infected neurons. AAV-DIO-hM3Dq-mCherry (**A**) or AAV-DIO-hM4Di-mCherry (**B** and **C**) was injected into the ARC of POMC-Cre mice and brain sections were immunostained for mCherry to detect infected neurons. Schematic sections correspond to immunostained sections. Regions that contained the cell bodies of mCherry+ neurons are indicated in red. Schematic sections are shown for 5 to 8 mice per condition as in Fig. 5D (A), Fig. 6D (B) and Fig. 6H (C) that were injected with CNO to test the effects of activating POMC neurons (A) or silencing POMC neurons when mice were restrained for 10 min (B) or 5 min (C). mCherry+ neurons are seen only in ARC.
